# Supplementary material for: Solar Photocatalytic Membranes: An Experimental and Artificial Neural Network Modeling Approach for Niflumic Acid Degradation
Source: Membranes (Basel). 2022 Aug 30;12(9):849. doi: 10.3390/membranes12090849 (PMC9504027; doi:10.3390/membranes12090849)
Supplement: Supplementary file 1 [file membranes-12-00849-s001.zip › membranes-1873260-supplementary.pdf]

# Solar photocatalytic degradation of niflumic acid using a $TiO_2$/PVDF–TrFE nanocomposite membrane: artificial neural network modeling

**L. Aoudjit[1†], H. Salazar[2, 3†*], D. Zioui[3], A.Sebti[3], P. M. Martins[4, 5], and S. Lanceros-Méndez[6, 7*]**

[1]*Unité de Développement des équipements Solaires, UDES /Centre de Développement des Energies Renouvelables, CDER, Bou Ismail, 42415, W. Tipaza, Algérie*
[2]*Centre/Department of Physics, University of Minho, Campus de Gualtar, 4710-057 Braga, Portugal*
[3]*Centre/Department of Chemistry, University of Minho, Campus de Gualtar, 4710-057 Braga, Portugal*
[4]*Centre of Molecular and Environmental Biology, University of Minho, Campus de Gualtar, 4710-057 Braga, Portugal*
[5]*Institute of Science and Innovation on Bio-Sustainability (IB-S), University of Minho, 4710-057, Braga, Portugal*
[6]*BCMaterials, Basque Centre for Materials, Applications and Nanostructures, UPV/EHU Science Park, 48940 Leioa, Spain*
[7]*IKERBASQUE, Basque Foundation for Science, 48013 Bilbao, Spain*

## Supporting Information

## 1. Supporting information for sub-chapter 3.

**Table S1.** Process variables and their operating range.

| Experimental variable | Operating range |
|---|---|
| Initial NFA concentration (mg/L) | 10 – 30 |
| pH | 3 - 9 |
| Irradiation time (h) | 0 -6 |
| Radiation intensity (W/m$^2$) | 269 - 925 |

## 2. Supporting information for sub-chapter 4.1.

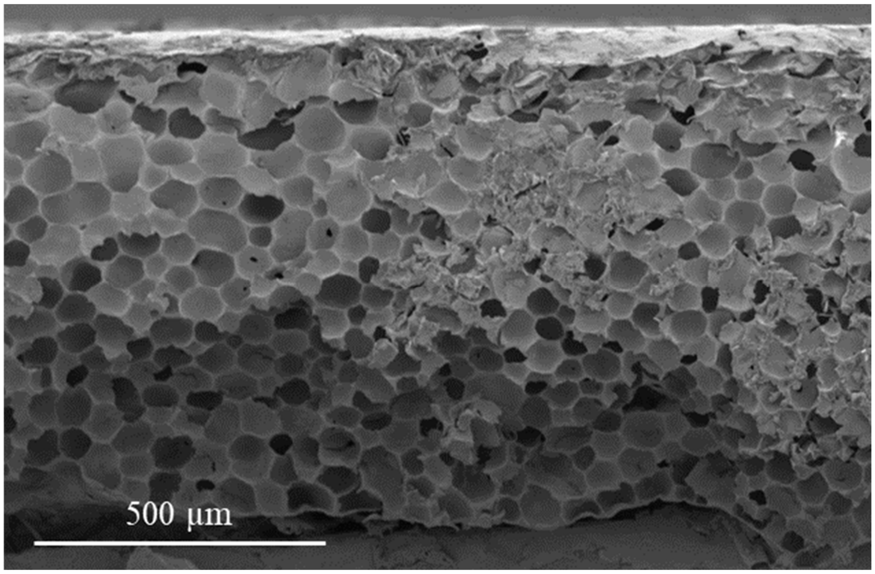


**Figure S1.** Representative SEM cross-section image of PVDF-TrFE membrane.

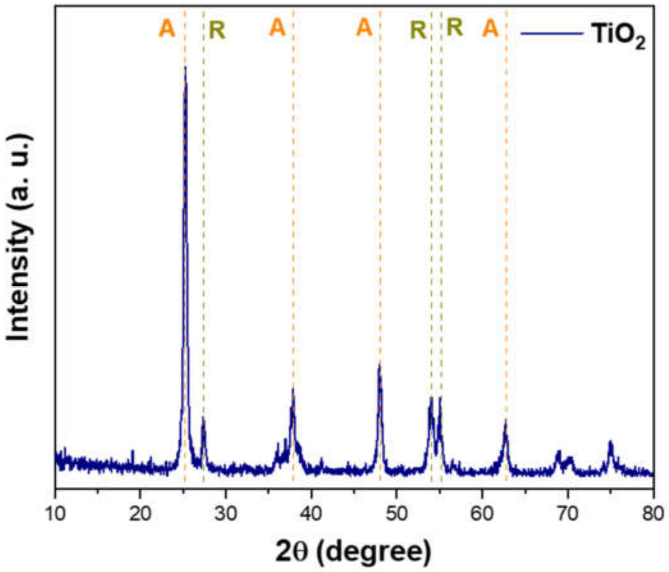


**Figure S2.** XRD pattern of $TiO_2$ nanoparticles.

## 3. Supporting information for sub-chapter 4.2.

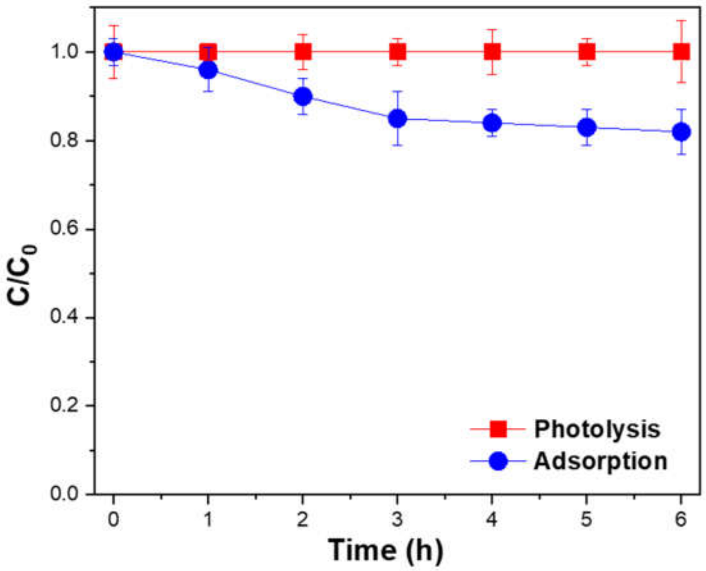


**Figure S3.** Effect of photolysis and adsorption processes on NFA degradation.

## 4. Supporting information for sub-chapter 4.2.1.

**Table S2.** Effect of initial NFA concentration on photocatalytic degradation efficiency and rate constant.

| $C_0$ (mg/L) | Efficiency (%) | Rate constant ($min^{-1}$) | $R^2$ |
|---|---|---|---|
| 10 | 91 | 0.28 | 0.93 |
| 20 | 76 | 0.17 | 0.95 |
| 30 | 59 | 0.11 | 0.94 |

## 5. Supporting information for sub-chapter 4.5.

**Table S3.** Weight and bias matrix of the ANN model.

| Weights and bias: Input layer – Hidden layer | | | | | | Weights and bias: Hidden layer – Output layer | | |
|---|---|---|---|---|---|---|---|---|
| Neuron | Inputs | | | | Bias | Neuron | Weights | Bias |
| | [NFA] (mg/L) | pH | Time (h) | Radiation intensity (W/m$^2$) | | | | |
| **1** | - 0,5071 | - 0,0503 | - 3,0541 | 0,1888 | - 2,7232 | **1** | - 0,5788 | - 0,3796 |
| **2** | 0,0970 | - 1,8525 | - 0,9105 | - 0,2183 | 2,1129 | **2** | 0,5938 | |
| **3** | 0,7826 | 1,1945 | 0,4955 | - 2,0058 | - 0,6591 | **3** | - 0,1363 | |
| **4** | - 1,9199 | - 0,3599 | 1,3549 | 1,1247 | 0,9924 | **4** | 0,1971 | |
| **5** | - 0,3115 | 1,0535 | - 1,2946 | 1,2275 | - 0,0473 | **5** | - 0,2787 | |
| **6** | 0,0291 | 2,8858 | 3,6648 | 1,1251 | - 1,9884 | **6** | 0,7312 | |
| **7** | - 1,6460 | 0,6069 | 0,3122 | - 1,5249 | - 1,8305 | **7** | 0,1854 | |
| **8** | - 1,3610 | - 1,8650 | 1,7781 | - 1,0967 | - 2,4857 | **8** | 0,0376 | |
